# An examination of inpatient medical record keeping in the Orthopaedic Department of Kilimanjaro Christian Medical Centre (KCMC), Moshi, Tanzania

**DOI:** 10.11604/pamj.2016.23.207.8083

**Published:** 2016-04-20

**Authors:** Alexander Conor Hollis, Samuel Robert Ebbs

**Affiliations:** 1 College of Medical and Dental Sciences, University of Birmingham, Edgbaston, Birmingham, B15 2TT, United Kingdom

**Keywords:** Tanzania, quality improvement, service evaluation, orthopaedics

## Abstract

**Introduction:**

There is a lack of published evidence examining the quality of patient notes in African healthcare settings. We aim to examine the completeness of the orthopaedic inpatient notes and begin development of a formal audit framework in a large Tanzanian Hospital.

**Methods:**

A retrospective review of 155 orthopaedic inpatient notes at Kilimanjaro Christian Medical Centre (KCMC) was conducted spanning 3 months. Notes were reviewed using an agreed data collection pro forma considering 3 main outcomes; i) quantity of complete entries, ii) percentage completeness of individual sections, iii) documentation of follow-up.

**Results: Primary outcome:**

8% (n = 13) of the inpatient documents were complete (10/10 sections). 11% (n = 17) of the inpatient documents had 9 of 10 sections completed. 30% (n = 46) of the inpatient documents had 8 of 10 sections completed. Therefore, 51% (n = 79) of inpatient entries had 7 or fewer sections filled in.

**Secondary outcome:**

Admission information and Demographics were both completed 88% (n = 137) of the time. History and the Examination sections were complete in 96% (n = 149) of cases. Investigations were complete in 77% (n = 119) and Diagnosis in 88% (n = 137). The Treatment section was complete 85% (n = 132) of the time and the Attending doctor 50% (n = 78). Procedures were 27% (n = 42) filled in while Summary of a day and Follow-up were 32% (n = 49) and 0% (n = 0) respectively.

**Tertiary outcome:**

Follow-up was not completed in any entries.

**Conclusion:**

There are a number of sections of the inpatient pro forma that remain inadequately completed. Regular auditing is essential for the continued progress in patient care.

## Introduction

There is some evidence of formal auditing of inpatient records in African countries, [1] however there is a paucity of such evidence in Tanzania. Several examples exist of inpatient record audits but these are regarding outcomes rather than the quality of the records themselves [2–3]. Nevertheless, evidence from heath systems in other developing countries has shown the need for audit to drive improved record keeping [4]. Studies from some developed countries have shown that auditing is a powerful tool for promoting and improving the quality of patient records [5–7]. There is also growing evidence that criteria based audits can be equally as effective in developing healthcare systems with poorer resources [8]. This shows the power of auditing in countries where perhaps there is not such a strong history and tradition of the evaluation of services. However, there remain a myriad of difficulties and barriers to conducting and implementing audits in developing healthcare systems which will be further explored later [9]. KCMC Hospital is a tertiary referral centre for northern Tanzania. It is a large teaching hospital which serves a population of around 11 million. According to the hospital annual report published in 2014 the top disease for the orthopaedic department was ‘Fracture of femur’ with 271 cases. The top killer disease was ‘Cervical injuries’. There were a total of 1415 admissions from January 2014 to December 2014. A total of 1102 procedures were documented and the most common procedure documented was'Surgical toilet’. All of this information was taken from a combination of hospital patient records and the in-house orthopaedic inpatient record books. The inpatient record books for the Orthopaedic Department of KCMC hospital were developed by Dr. T Rodgers in 2013 as a way of improving record keeping and data collection in the department. They serve as a dynamic source of patient information, which can be used both in optimizing patient care and conducting research. The inpatient books are currently used by the department to perform research and were used heavily in the 2014 annual report published by KCMC. As no formal audit of these books has been undertaken it is impossible to evaluate the reliability of this data and the utility of these books as a tool for research. A clinical audit will verify the quality of the record keeping and therefore validate past and future research taken from the data.

**Aims:** This audit aims to assess to what extent each admission has been recorded in keeping with the pro forma in the document. We developed three outcome measures to assess the completion of the books: -Primary outcome: The number of inpatient entries completely filled in. -Secondary outcome: The percentage completeness of individual sections of the entry. -Tertiary outcome: The documentation of follow up and progress on the ward.

## Methods

A retrospective analysis of 155 consecutive patients included in the inpatient books between the 19th March 2015 and 6th May 2015 was performed. Any patient admitted during this period was included. We excluded all records that had been either crossed out or left blank. There were no other formal exclusion criteria. In order to assess the records we developed our own Medical Records Review tool (MMR). This questionnaire was then used to assess the completeness of the records. We assessed the completeness of documentation by splitting it into a number of sections. These included: Admission information (date of admission and serial number), demographics (age, sex and patient number), history, examination, investigations, diagnosis, and treatment, attending doctor, procedures, summary-of-a-day and follow-up. Our standard for completeness was agreed upon both with the current head of the department, Dr Mandari and the designer of the books, Dr Temu. We considered an entry in a section to be complete if the following criteria were filled in: -admission information: serial number and date of admission, -demographics: age, sex and hospital number, -history: Presence of documentation in correct section, -examination: Presence of documentation in correct section, -investigations: Presence of documentation in correct section, -diagnosis: Presence of documentation in correct section, -treatment: Presence of documentation in correct section, -attending doctor: Named doctor documented, -procedures: Procedures noted in correct section, -summary-of-a-day: 1^st^,2 ^nd^, and 3 ^rd^, named doctor on-call. Follow-up was measured but excluded from the final primary outcome analysis as it is often completed at a different time to the rest of the inpatient book. We agreed to consider this as a separate outcome. An inpatient sheet was considered satisfactory if all 10 sections listed above were complete. One important point to note it that ‘complete’ was presence of documentation. In the case of missing information, if this had been indicated e.g with ‘N/A'or an obvious line or cross through the section then this would be considered complete.

## Results

A total number of 155 patient entries were analysed according to our medical records review pro forma.

**Figure 1:**
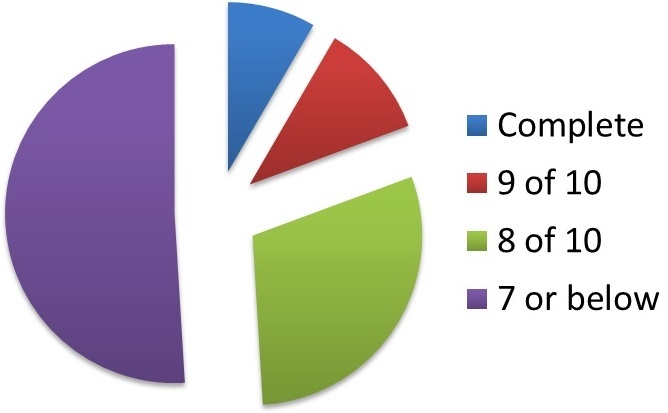
Degree of completeness of each pro forma

**Primary outcome:** 8% (n = 13) of the inpatient documents were complete (10/10 sections). 11% (n = 17) of the inpatient documents had 9 of 10 sections completed. 30% (n = 46) of the inpatient documents had 8 of 10 sections completed. Therefore, 51% of inpatient entries had 7 or fewer sections adequately filled in (Figure 1).

**Figure 2:**
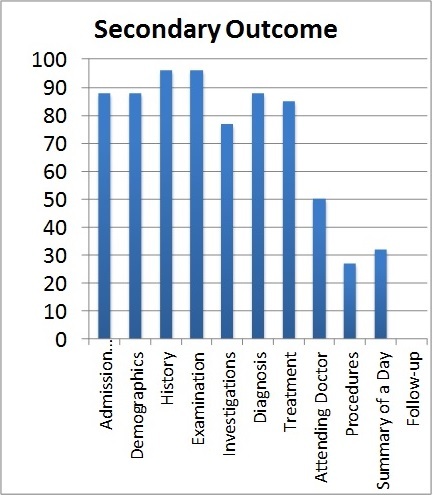
Extent to which each category in the pro forma was completed

**Secondary outcome:** the extent to which each category in the inpatient entry was filled out is summarized below: - admission information: 88%, n = 137, -demographics: 88%, n = 137, -history: 96%, n = 149, -examination: 96%, n = 149, -investigations: 77%, n = 119, -diagnosis: 88%, n = 137, -treatment: 85%, n = 132, -attending doctor: 50%, n = 78, -procedures: 27%, n = 42, -summary of a day: 32%, n = 49, -follow-up: 0%, n = 0 (Figure 2). Tertiary outcome: Follow up was recorded in 0 of the 155 records that were assessed.

**Summary of results:** Over 50% of the inpatient entries had 3 or more sections that were considered incomplete. With regards to the individual sections, attending doctor, procedures and summary-of-a-day were the most poorly completed. Follow-up was not recorded in any of the 155 inpatient entries analysed.

## Discussion

There is clearly a large discrepancy between the standard of record keeping in these inpatient books and the standards held by many developed healthcare systems. There are a number of reasons for such a discrepancy, some of which we will now address. Staff shortages are one major problem that was identified. The department has 3 specialists and 15 residents with 4 intern doctors. The 4 intern doctors have the task of clerking all new admissions (1415 in 2014). The department itself also regularly functions over capacity with stretchers and corridors being used to accommodate overflow. There is also a rapid turnover of interns with each intern completing a 4 week attachment with the department. Crucially, a culture of precise record keeping is lacking [9]. In the UK and much of the developed world there is a well-established culture of auditing and sound record keeping, partially due to the pressure of medico-legal issues. However many of these patients come from poor, impoverished backgrounds and often struggle to afford medical care, let alone professional legal advice. As a result, there is far less pressure from legal proceedings and the drive to document all medical procedures is correspondingly low. Institutional commitment is also a key driver of change in practice. Currently there is no designated clinical audit department at KCMC hospital. Regular commitment to audit needs to become a part of both evaluating outcomes and services and also on a more basic level in evaluating the quality of record keeping. It is also important to note that missing patient data is far more of a problem in the developing world than the modern health care systems [9]. Therefore any audit that is undertaken must account for the inevitable lack of some demographic or clinical data. Studies in other African countries namely Botswana [9] have aimed to identify the obstacles to conducting and implementing clinical audits in developing countries. This would be a useful study to undertake in Tanzania as they will likely share many of the same issues.

There are three main types of bias in any clinical research; pre-trial bias, bias during the trial and bias after the trial. We aimed to remove pre-trial bias by defining specific outcomes and inclusion criteria. The main selection bias we must account for is that we did not cross reference admission data with patient records. This means that patients may have been admitted and not recorded into the inpatient book. Another problem exists insofar as there is only one inpatient book for the orthopaedic department. As a result, patients are often recorded on separate pieces of paper and never written up into the book. Regarding bias that occurred during the trial, we aimed to use objective measures to minimize this. We were not measuring quality of clinical documentation merely the objective presence of documentation. Transfer bias was the biggest issue that we faced due to lack of follow up. The fact that none of the records we reviewed had follow-up recorded implies that this section was simply not being filled out rather than not being recorded. We discovered that the inpatient book would be filled in by the intern on call and that follow up tended to be put into the patient notes either at ward review or outpatient appointments. This highlights a fundamental problem with the inpatient book, and paper notes generally. As it is a physical entity, the book cannot be updated in outpatient appointments whilst still being available for the clerking intern. Therefore it is imperative that once follow up has been done, this is immediately recorded into the inpatient book by the attending doctor. The reliability of the study is also difficult to quantify as so far it has only been carried out on a single sample of the inpatient books. The second cycle of the audit must be completed to truly determine whether or not the study is reliable. The second cycle will be carried out in six months’ time by a designated intern and will use the same study design and pro forma. Once the cycle has been closed then a complete audit report may be written. To summarise, the data cannot be accurately generalized beyond the inpatient record books as this is a niche area for a specific department. Further prospective research is needed to evaluate the current standard of medical note keeping. However the results are in keeping with the literature [8] as they have found an inadequacy in the documenting of patient notes.

**Recommendations:** Following the completion of the audit it was presented to the entire department as part of the morning report. The following recommendations were made to the department: -The importance of correct record keeping should be stressed to the interns both from a patient-care and research perspective. -The interns responsible for filling in the inpatient books should be taught how to adequately fill in the books according to the agreed standard. -6-monthly auditing of the inpatient books should be a mandatory part of the interns’ training. -A weekly check of the book by seniors to assure that it is being completed. -If information is missing, it should be clearly stated that this is absent as opposed to leaving sections blank. -In addition, sections in the pro-forma should be filled in according to their title, to maintain clarity of notes. -It should be the responsibility of the discharging doctor to return to the inpatient book and complete the required section on ‘follow-up’. This should also be signed by the respective physician. -The quality of the entries should be graded according to agreed criteria by the heads of department and medical school. This can they form an objective assessment of the interns progress on the rotation. These recommendations were agreed within the department after a discussion within the department following the presentation. One of the key development points is the potential inclusion of the inpatient books into the intern's yearly assessment. This would incentivise and reward the interns for completing the data in both the inpatient book and medical notes. The 6-monthly audits of the inpatient books are also a crucial part of the recommendations as they will allow the audit cycle to be completed and allow assessment of the extent to which the recommendations have been taken on board.

**Future:** This audit has provided questions and begs for further auditing and research. First and foremost, the cycle of this audit must be completed to evaluate its effectiveness. After this, a regular audit system should be implemented to carry on the momentum of these changes. Another key area to audit would be the content and quality of the notes themselves. This would involve proper coding and documenting of medical conditions. In order to make long lasting changes the hospital as a whole will need to set up a dedicated audit office. Accurate and effective medical record keeping comprises a key component if this auditing system is to thrive. Improving record keeping is vitally important not just to orthopedics but to all departments of KCMC.

## Conclusion

This audit of inpatient record books has the potential to directly influence and improve clinical practice in this centre. Clearly, there are significant problems with the record keeping in the inpatients books and every effort has been made both to identify and rectify the reasons for this. Recommendations have been made to improve future record keeping. However it will only be once a culture of increased priority of record keeping develops that changes will be fully appreciated. It is also vital that the 6 monthly audit is completed so that the longer-term outcomes of this audit may be understood.

### What is known about this topic

- There is some evidence of formal auditing of inpatient records in African countries, however there is a paucity of such evidence in Tanzania.
- Several examples exist of inpatient record audits but these are regarding outcomes rather than the quality of the records themselves.
- Nevertheless, evidence from heath systems in other developing countries has shown the need for audit to drive improved record keeping. What this study adds.

### What this study adds

- This audit of inpatient record books has the potential to directly influence and improve clinical practice in this centre.
- Clearly, there are significant problems with the record keeping in the inpatients books and every effort has been made both to identify and rectify the reasons for this.
